# *Agaricus Subrufescens* ameliorates ovarian dysfunction and regulates altered biochemical parameters in rats with Letrozole induced polycystic ovarian syndrome

**DOI:** 10.1186/s13048-023-01311-1

**Published:** 2023-11-22

**Authors:** Sarad Pawar Naik Bukke, Bharghava Bhushan Rao Pathange, Srikanth Kumar Karumanchi, Jalaiah Marri, Revathi Boyina, Kavya Rachamsetty, Bhargavi Manchikalapati, Saidi Odoma, Bello Hussaini

**Affiliations:** 1 Department of Pharmaceutics and Pharmaceutical Technology, Kampala International University, Western Campus, P.O. Box 71, Ishaka-Bushenyi, Uganda; 2 Department of Pharmaceutics, AM Reddy Memorial College of Pharmacy, Narasaraopet, Andhra Pradesh India; 3 Department of Pharmaceutical Chemistry, DKSS Institute of Pharmaceutical Science and Research for Girls, Swami Chincholi-413130, Pune District, Maharashtra India; 4 Department of Pharmacology, QIS College of Pharmacy, Ongole, Andhra Pradesh India; 5 Department of Pharmacy Practice, QIS College of Pharmacy, Ongole, Andhra Pradesh India; 6 Department of Pharmacology and Toxicology, Kampala International University, Western Campus, P.O. Box 71, Ishaka-Bushenyi, Uganda

**Keywords:** *Agaricus Subrufescens*, Polycystic ovarian syndrome, Letrozole, Histopathological study, Vaginal smear, Estrogen, Blood glucose level

## Abstract

**Objective:**

The objective of this study is to investigate the effects of an ethanolic extract derived from *Agaricus subrufescens* on rat models exhibiting Polycystic Ovarian Syndrome (PCOS) induced by Letrozole.

**Methods:**

A total of thirty female Wistar rats were divided into five groups, each consisting of six rats. The negative control group was administered a volume of 1 mL of a 0.5% solution of carboxy methylcellulose (CMC). Letrozole (1 mg/kg) was administered to additional groups for a duration of 21 days in order to induce polycystic ovary syndrome (PCOS). Animals designated as positive controls were euthanized on the 22nd day. Both the test group and the standard group were subjected to treatment from the 22nd day to the 36th day. The experimental group was administered ethanolic extract of *Agaricus subrufescens* at doses of 200 mg/kg and 400 mg/kg p.o, while the control group received clomiphene citrate at a dose of 1 mg/kg. The study observed various physiological markers in individuals with polycystic ovarian disease, including estimated blood glucose levels, total cholesterol levels, triglyceride levels, and hormonal fluctuations such as increased testosterone and estrogen levels, as well as decreased progesterone levels. The presence of menstrual irregularities was confirmed through the examination of vaginal smears and histopathological changes in the ovaries.

**Results:**

The consumption of *Agaricus subrufescens* was found to have a significant impact on various physiological parameters, including blood glucose levels, testosterone levels, anovulation, and menstrual irregularity. All therapeutic interventions significantly normalized the levels of serum glutamic-oxaloacetic transaminase (SGOT) and serum glutamic-pyruvic transaminase (SGPT). The rats with polycystic ovary syndrome (PCOS) that were induced by Letrozole exhibited increased levels of urea and creatinine. The findings of this study indicate that the administration of *Agaricus subrufescens* therapy has a protective effect on renal function, as evidenced by a reduction in serum levels of urea and creatinine. In rats with polycystic ovary syndrome (PCOS) induced by Letrozole, the inhibition of hepatic synthesis, promotion of ovarian follicle immaturity, and elevation of androgen secretions result in an increase in the weight of the liver and ovaries. The weight of endocrine organs exhibited a decrease across all treatment groups. The histopathological examination of PCOS specimens revealed an increased presence of cysts and theca lutein cells. The group of rats with polycystic ovary syndrome (PCOS) that did not receive treatment exhibited a higher number of cysts compared to the groups that received treatment.

**Conclusion:**

This study demonstrated that the administration of Letrozole orally resulted in the development of polycystic ovarian disease. The results indicated heightened levels of blood glucose, total cholesterol, and triglycerides, as well as alterations in hormone levels such as increased testosterone and estrogen, and decreased progesterone. These hormonal changes were accompanied by menstrual irregularities, which were confirmed through the examination of vaginal smears and histopathological analysis of the ovaries in the control group with polycystic ovarian disease. The treatment groups that received *Agaricus subrufescens* exhibited a decrease in blood glucose, total cholesterol, and testosterone levels.

## Introduction

Polycystic ovary syndrome (PCOS) is a prevalent endocrine disorder characterized by the presence of multiple cysts (fluid-filled sacs) on the ovaries, hyperandrogenism, impaired ovulation, abdominal obesity, and various metabolic abnormalities. Polycystic ovary syndrome (PCOS) is associated with various reproductive and endocrine abnormalities, including infertility, menstrual irregularities, hirsutism, and elevated testosterone levels [1, 2]. PCOS causes acne, acanthosis nigricans, insulin insensitivity, hyperandrogenism, and persistent anovulation [3–6]. The enduring consequences encompass various health conditions such as cancer, type II diabetes, dyslipidemia, hypertension, and cardiovascular disease. The reproductive and metabolic effects of polycystic ovary syndrome (PCOS) can be reversed through weight loss and increased physical activity. Polycystic ovary syndrome (PCOS) may be attributed to lipid imbalance, oxidative stress, insulin resistance, and genetic factors [7, 8]. The etiology of this condition can be attributed to dysregulation of the hypothalamic-pituitary axis, insulin abnormalities, and ovarian dysfunction. There exists a correlation between insulin resistance and obesity [9]. Insulin resistance has been observed to enhance the production of androgens in cells through the stimulation of luteinizing hormone, while concurrently impeding the synthesis of sex hormone binding globulin (SHBG) in liver cells. The study found that there is a reduction in follicular stimulating hormones within granulose cells, which leads to the cessation of follicular development [10].

Both insulin-resistant and non-insulin-resistant polycystic ovary syndrome (PCOS) are prevalent in the population. Insulin-resistant polycystic ovary syndrome (PCOS), also referred to as "Type 1 PCOS," is responsible for the majority of PCOS symptoms. The observed physiological manifestations include an increase in body weight, disruptions in ovulation, the development of excessive facial hair, hair loss, and the presence of acne. Insulin resistance in polycystic ovary syndrome (PCOS) is associated with the development of hyperglycemia and elevated levels of testosterone. Insulin resistance exerts an impact on the hypothalamus pituitary ovarian axis, leading to an upregulation of androgen production in ca cells and a downregulation of androgen production in liver cells. This dysregulation results in the manifestation of hyperandrogenism, anovulation, and polycystic ovarian syndrome. Non-insulin-resistant polycystic ovary syndrome (PCOS) can be attributed to various factors, including vitamin D deficiency, iodine deficiency, hormone-disrupting contaminants, thyroid disease, and adrenal stress. Anti-diabetic medications are ineffective in treating polycystic ovary syndrome (PCOS) in women who do not exhibit insulin resistance, and they do not assist in the reduction of weight caused by hormonal imbalances. Natural remedies. In conjunction with refraining from dairy consumption, individuals may consider incorporating iodine, vitamin D, magnesium, zinc, and herbs known for their testosterone-lowering properties into their dietary regimen. The administration of natural progesterone has been found to have a regulatory effect on hormone levels and the process of ovulation [11–15]. Approximately 4–12% of women in the United States who are of reproductive age are affected by Polycystic Ovary Syndrome (PCOS). According to existing research, it has been found that approximately 10% of women are affected by Polycystic Ovary Syndrome (PCOS). The prevalence of polycystic ovary syndrome (PCOS) among European women ranges from 6.5% to 8%. The percentage range observed is from 2.2% to 26%. Despite the absence of empirical evidence, it is widely posited among experts that approximately 10% of women in India are affected by Polycystic Ovary Syndrome (PCOS). Polycystic ovary syndrome (PCOS) is a multifaceted endocrine disorder characterized by genetic complexity, an elusive etiology, and intricate pathogenesis. The symptoms of polycystic ovary syndrome (PCOS) can potentially coincide with the natural physiological changes that occur during the two-year period following the onset of menstruation, thereby causing a delay in the clinical identification and diagnosis of the condition. Women who have a genetic predisposition to polycystic ovary syndrome (PCOS) and are slender in body composition may experience weight gain. The prevalence rates of hirsutism, acne, female pattern hair loss, acanthosis nigricans, seborrhea, striae, and acrochordons were reported as 78%, 48%, 31%, 30%, 29%, 13%, and 9% respectively [16–18]. Polycystic ovaries are characterized by the presence of multiple small follicles, typically ranging in size from 4 to 9 mm in diameter. Follicles of small size are incapable of undergoing ovulation. Dysregulation of estrogen, progesterone, luteinizing hormone (LH), and follicle-stimulating hormone (FSH) levels. The adrenal glands and ovaries are responsible for the production of androgens. DHEA, DHEAS, testosterone, and androstenedione are classified as androgens. There is evidence to suggest that women with polycystic ovary syndrome (PCOS) may experience elevated levels of LH and insulin, which in turn can contribute to increased androgen production [19, 20].

The key goal of this study is to evaluate the effectiveness of *Agaricus subrufescens* in treating polycystic ovary syndrome (PCOS) through the assessment of blood glucose levels, identification of menstrual cycle irregularities, biochemical analysis, hormonal evaluation, and examination of follicular cyst formation in rats with Letrozole-induced PCOS.

## Materials & methods

### Plant profile

Figure 1.

**Fig. 1:**
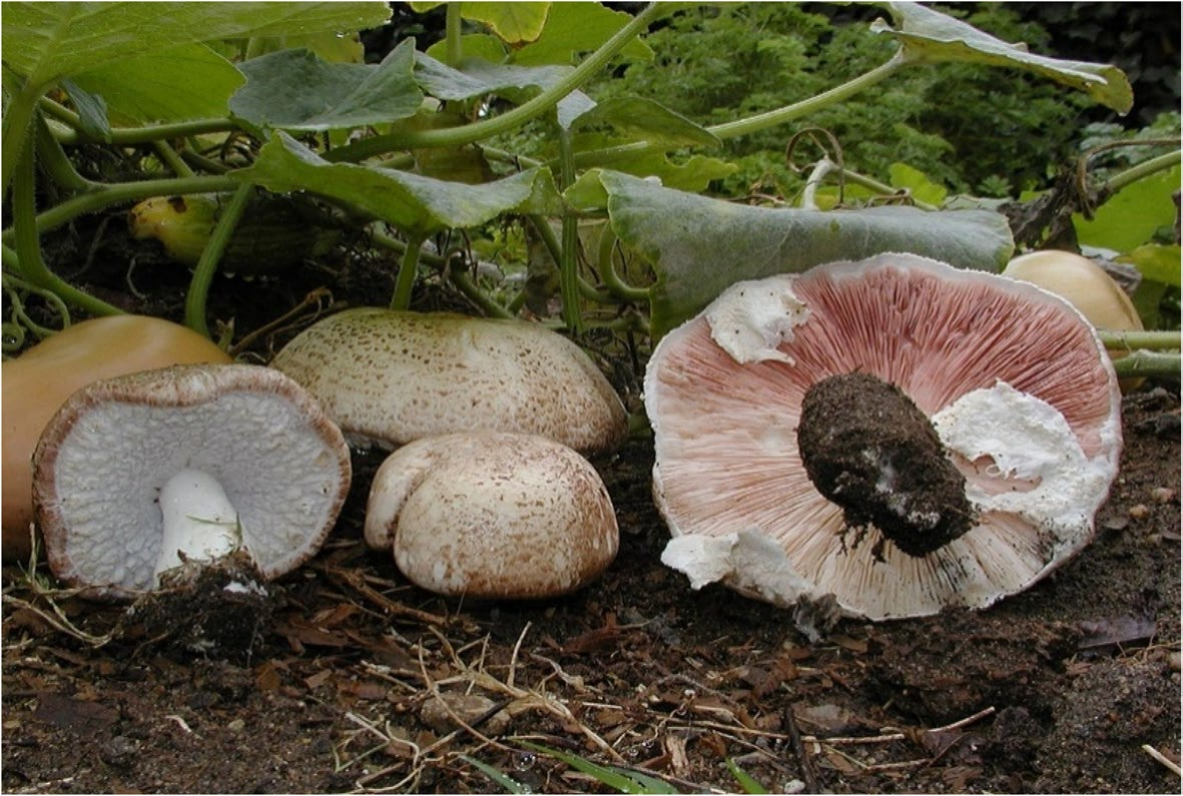
Agaricus Subrufescens

*Agaricus subrufescens,* a member of the Agaricaceae family, is known by various names such as almond mushroom, mushroom of the sun, God's mushroom, mushroom of life, royal sun agaricus, jisongrong, himematsutake, and others. *Agaricus subrufescens* is a delectable edible with a pleasant aroma reminiscent of almonds. The fungus is recognized in both traditional and alternative medicine as a medicinal mushroom possessing anti-cancer properties. The efficacy and safety of Agaricus mushrooms as a food source, dietary supplement, or medicinal product have not been sufficiently evaluated [21, 22].

#### Plant collection, drying and authentication

The material was collected and desiccated on filter paper sheets under shaded conditions at ambient temperature. The plant underwent desiccation within a period of one month. Once more, the substance is dehydrated and pulverized. Fresh specimens of *Agaricus subrufescens* were obtained from the Nallamalla Forest in the state of Andhra Pradesh. The *Agaricus subrufescens* was validated and authenticated by Dr. Ramadevi, an Associate Professor at Dr. Y.S.R. Horticulture University in West Godavari, Andhra Pradesh, India.

#### Extraction procedure

The dried and powdered substance was subjected to ethanol extraction using the Soxhlet apparatus for a duration of 18 h. The Soxhlet apparatus was employed to extract a powdered substance by dissolving it in a solution of ethanol and water in a ratio of 1:3. The extract obtained from the round-bottom flask was subjected to evaporation until complete dryness, resulting in the production of a yellowish-brown *Agaricus subrufescens* extract (EAS). This extract was subsequently stored in a container that ensured airtight conditions. The crude extract of the EAS was stored under ambient conditions and subjected to phytochemical analysis in order to identify plant secondary metabolites [23].

#### Stock solution preparation

Using CMC as a diluting solvent, a 1 mg/ml solution was prepared.

#### Test dose preparation

200 mg/ml and 400 mg/ml solutions prepared from the aforementioned 1 mg/ml solution. Animals received doses in accordance with their body weight.

#### Standard dose preparation

CMC was used as a diluting solvent to prepare 1 mg/ml solution of clomifene citrate. Animals received doses in accordance with their body weight.

### Phytochemical screening

Several qualitative tests were done to check the existence of phytochemical analysis in the ethanolic crude extract of *Agaricus subrufescens* (EAS) [24, 25].

#### Acute toxicity studies

The acute toxicity studies adhered to the guidelines outlined by the Organization for Economic Cooperation and Development (OECD) in their standard 425, as prescribed by the Committee for the Purpose of Control and Supervision of Experiments on Animals (CPCSEA). The animals abstained from consuming any food for a duration of 4 h, relying solely on water. During a 14-day period, mice were administered ethanolic extract of *Agaricus subrufescens* at doses of 1000 and 2000 mg/kg. The mice were subsequently observed for mortality as well as any physical and behavioral abnormalities. The experiments were authorized by the Institutional Animal Ethical Committee [26].

#### Animal groups design

- Group-I: Normal control (Received 2 ml distilled water orally chow diet).
- Group-II: Letrozole (LTZ) control; Scarified on day 22.
- Group-III: LTZ + clomiphene citrate (STD); Received 1.0 mg/kg body weight dose of clomiphene citrate for 15 days post Letrozole induction.
- Group-IV: LTZ + EAS (Treatment-1); Received 200 mg/kg body weight dose of clomiphene citrate for 15 days post Letrozole induction.
- Group-V: LTZ + EAS (Treatment-2); Received 400 mg/kg body weight dose of Clomiphene citrate for 15 days post Letrozole induction.

#### Study design

Mahaveer Enterprises supplied 180–220 g Wister albino female rats, which were all kept in conditions of 27 ± 2 °C, 80 ± 10% humidity, and a 12-h light/dark cycle. The subjects were provided with a standard rat diet and were given unrestricted access to water. Each case contained three rats housed on husk bedding. The trial commenced following a period of seven days dedicated to acclimating. Female rats were administered Letrozole at a dosage of 1.0 mg/kg, dissolved in a vehicle consisting of 0.25% CMC (2 ml/kg body weight), on a daily basis for a period of 21 days in order to induce the development of polycystic ovary. On the 22nd day of the experiment, mice in the PCOS control group were subjected to anesthesia using diethyl ether. Blood samples were collected from the retro orbital region through puncturing to determine levels of blood glucose, estrogen, progesterone, and total cholesterol. The weights of uteruses, ovaries, kidneys, livers, and hearts were measured subsequent to the slaughter of animals. The animals in the control group were administered Clomiphene citrate for a duration of 15 days, while the experimental group received different dosages of plant extract. On the 36th day of the experiment, the animals underwent anesthesia using diethyl ether and were subjected to retro orbital puncture in order to measure blood glucose, estrogen, progesterone, and total cholesterol levels. Subsequently, the animals were sacrificed and their uterus, ovaries, kidneys, liver, and heart were weighed [6, 27, 28].

#### Preparation of vaginal smear

The assessment of ovarian function through the vaginal smear method is contingent upon the response of the vaginal epithelium to ovarian hormones. The response of the vaginal epithelium to ovarian hormones was assessed through daily smears conducted at 10am. Every day, smears were collected at 10am. Female animals were anesthetized with diethyl ether and positioned on their ventral surfaces. Moisten a cotton-tipped applicator with isotonic saline solution. The rat's vaginal swab was meticulously rotated in a clockwise direction following its insertion to a predetermined depth. With caution, I proceeded to collect the vaginal swab and gently transferred it onto a glass slide under microscopic observation. The glass slide experienced discoloration as a result of the drying process applied to crystal violet or methylene blue. The glass slide was subjected to a meticulous washing and drying process using water for a duration of one minute. The cells were examined using 10X light microscopes [29].

#### Estimation of biochemical parameters

On the 22nd day, the mice in the PCOS-induced group were subjected to anesthesia using diethyl ether. On the 36th day, anesthesia was administered to all groups of mice. Blood samples were obtained from the retroorbital plexus, and the serum was isolated through the process of centrifugation. The centrifugation was performed on whole blood without the use of anticoagulants, at a speed of 3000 revolutions per minute (rpm) for duration of 10 min. The levels of blood glucose, total cholesterol, triglycerides, SGOT, SGPT, urea, and creatinine were determined using standard laboratory techniques [30, 31].

#### Hormonal estimation, organs weight measurement, body weight monitoring

The levels of serum testosterone, oestrogen, and progesterone were quantified using enzyme immunoassay kits [32]. Following the completion of the study, the animals were euthanized and their liver, kidney, heart, uterus, and ovary were collected, subjected to thorough examination, and subsequently weighed. The animals were weighed each morning using a weighing balance until the conclusion of the experiment [4]. Every morning, feed intake was subtracted.

### Histopathology

Ovaries were dissected shortly after scarification. The specimens were subsequently fixed in formalin for a duration of 16 h. Following the fixation process, the samples underwent dehydration by submerging them in a sequence of alcoholic solutions with progressively higher concentrations, typically consisting of 70%, 90%, and 100% alcohol, for a duration of 15 min per solution. Following a 20-min immersion in xylene, the samples were subsequently transferred into a tissue embedding cassette and enveloped with molten, unadulterated paraffin wax. A duration of 20 min at a temperature of 20 °C. The samples were prepared by being sliced into sections with a thickness of at least 2 μm and subsequently observed using a microscope.

## Results & discussion

The ethanolic extract of *Agaricus subrufescens* was subjected to phytochemical screening, which identified the presence of alkaloids, carbohydrates, glycosides, tannins/polyphenols, phytosterols, triterpinoids, flavonoids, saponins, and proteins. Table 1.

**Table 1:** Phytochemical investigation of *Agaricus Subrufescens*

**S. No**	**Test**	**Presence**
1	Alkaloids	+
2	Carbohydrates	+
3	Glycosides	+
4	Tannins or Phenolic Compounds	+
5	Phytosterols and Triterpennoids	+
6	Flavonoids	+
7	Saponins	+
8	Proteins	+

The administration of a single oral dose of at a dosage of 1 mg/kg body weight in female rats resulted in the development of polycystic ovarian syndrome. The animals exhibit irregular estrus cycles, anovulation, hormonal imbalances, abnormal follicular development, hyperlipidemia, and hyperglycemia. The results of this study demonstrated the induction of polycystic ovary syndrome (PCOS). The management of polycystic ovary syndrome (PCOS) through the use of Letrozole has been found to result in weight reduction. Significant weight changes were observed in the treatment groups when compared to both the normal and PCOS control groups. The impact of natural healing on weight was found to be comparable to that of both normal control and PCOS control groups. The administration of Letrozole effectively regulated polycystic ovary syndrome (PCOS) and resulted in a reduction in the consumption of feed. All experimental groups exhibited greater levels of feed consumption compared to both the normal control group and the PCOS control group. The feed intake of individuals undergoing natural recovery was found to be comparable to that of individuals in the normal and polycystic ovary syndrome (PCOS) control groups. The diagnosis of polycystic ovary syndrome (PCOS) was predicated upon the utilization of hormonal testing. Polycystic ovary syndrome (PCOS) is associated with an elevation in estrogen levels. The rats with Letrozole-induced polycystic ovary syndrome (PCOS) exhibited elevated levels of blood estrogen and testosterone, while experiencing decreased levels of progesterone compared to the PCOS control group. Letrozole exerts its effects by augmenting the pituitary gland's responsiveness to gonadotropin-releasing hormone (GnRH), resulting in elevated levels of luteinizing hormone (LH) and insulin. These hormonal changes predominantly exacerbate the existing abnormalities in steroidogenesis. Hyperandrogenism is a condition that arises due to an excessive amount of androgens. *Agaricus subrufescens* subjected them to normalization.

**Fig. 2:**
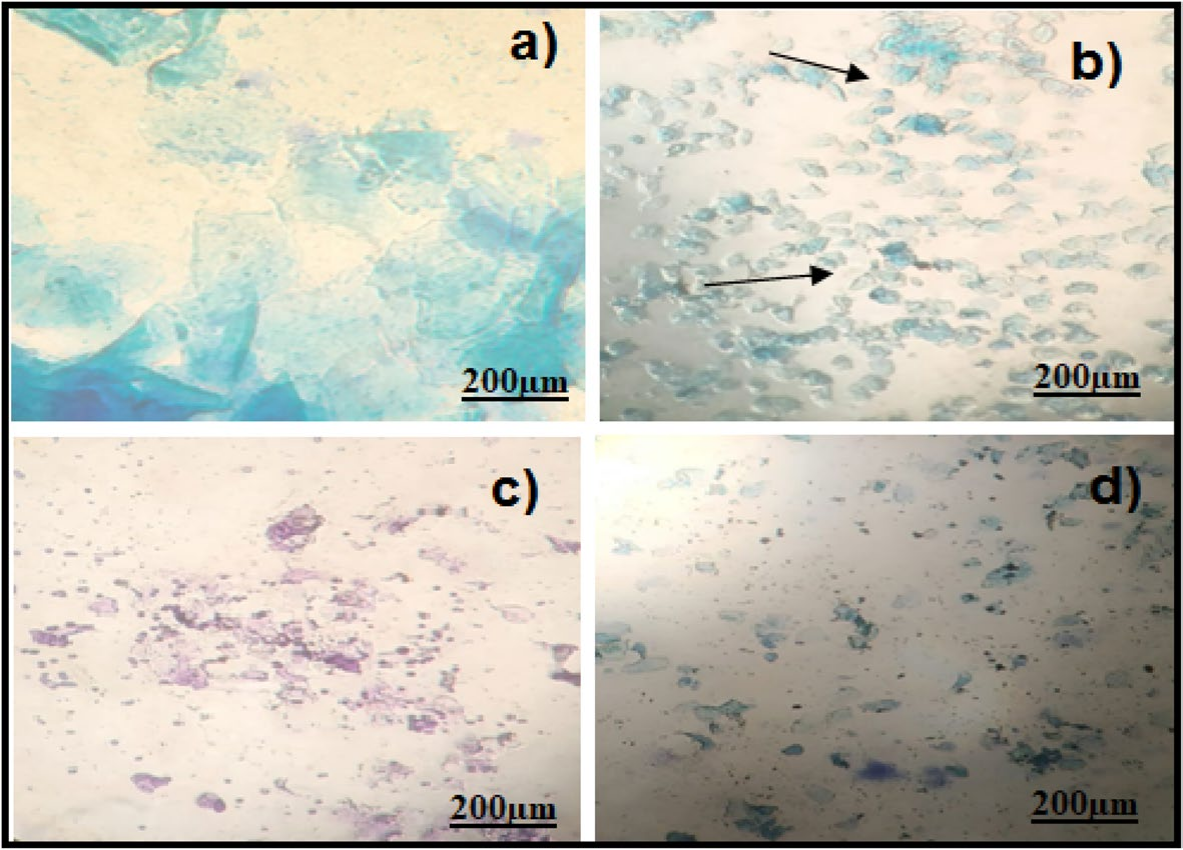
Group-I animal cells in the estrus cycle under a microscope; **a**) Proestrous **b**) Estrus **c**) Metaestrus **d**) Diestrus

**Fig. 3:**
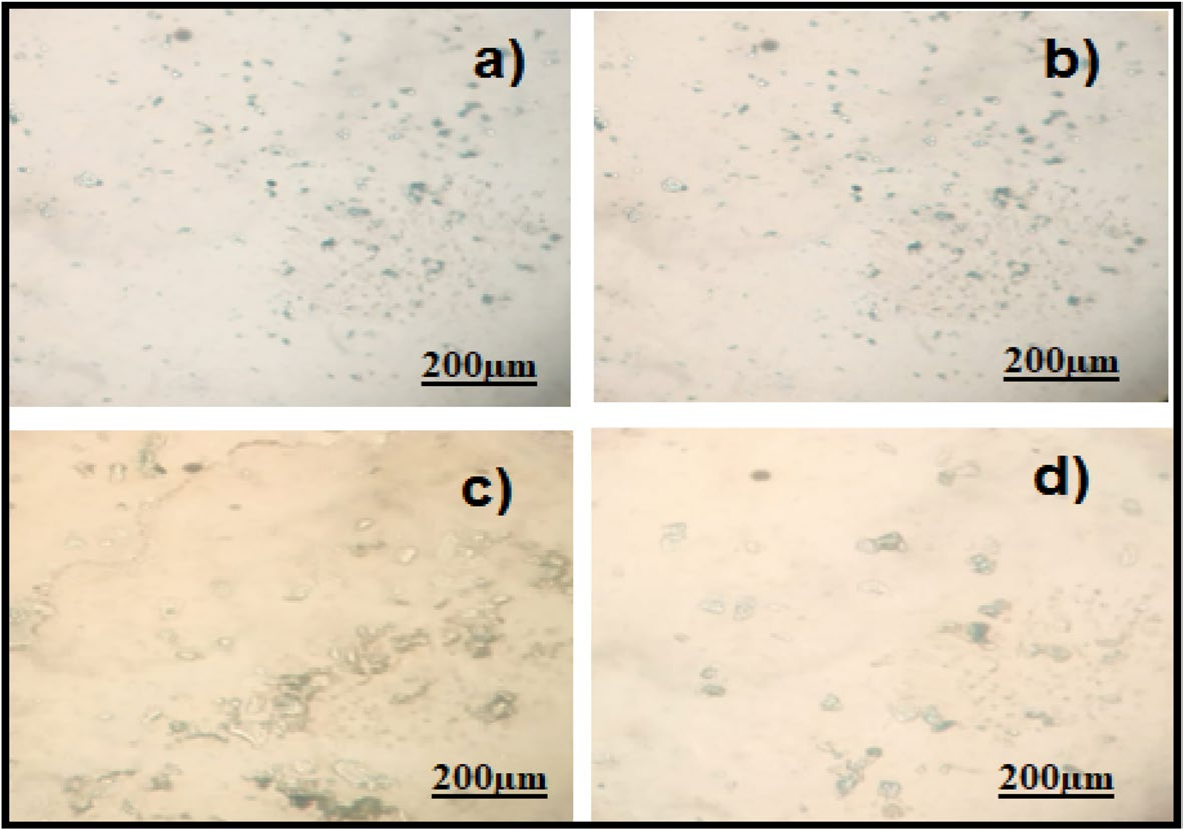
Letrozole induced animal cells in the estrus cycle under a microscope; **a**) Proestrous **b**) Estrus **c**) Metaestrus **d**) Diestrus

**Fig. 4:**
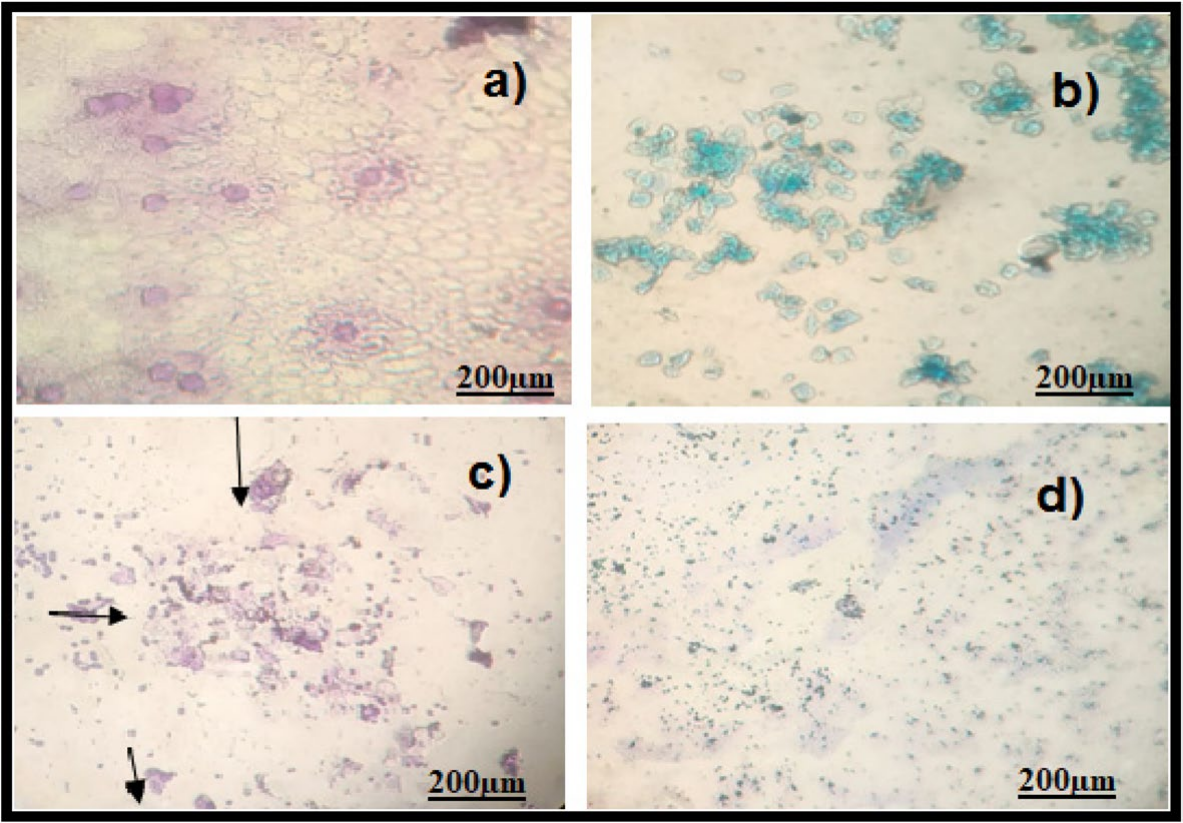
Treatment group animal cells in the estrus cycle under a microscope; **a**) Proestrous **b**) Estrus **c**) Metaestrus **d**) Diestrus

**Fig. 5:**
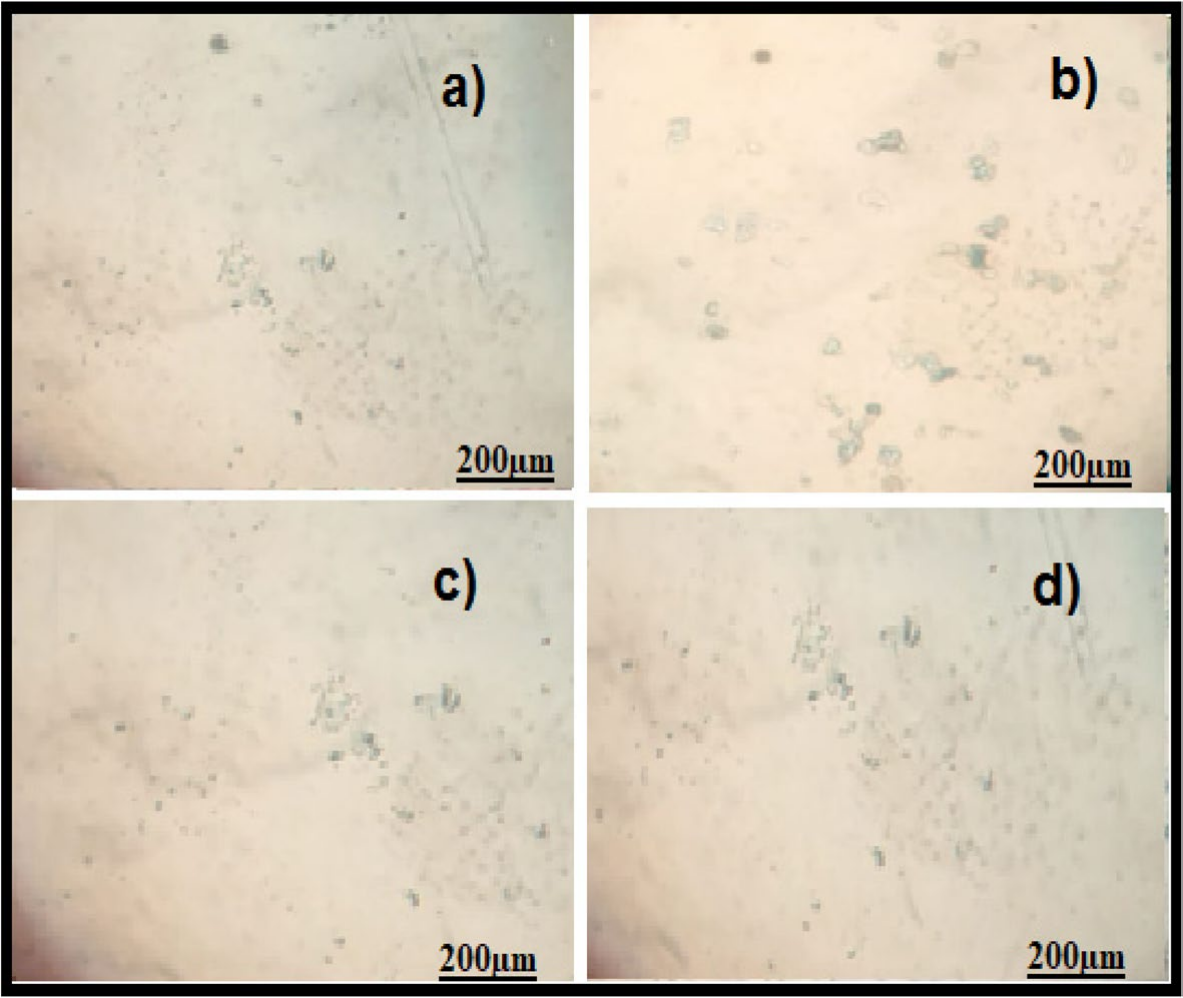
Natural recovery group animal cells in the estrus cycle under a microscope; **a**) Proestrous **b**) Estrus **c**) Metaestrus **d**) Diestrus

**Fig. 6:**
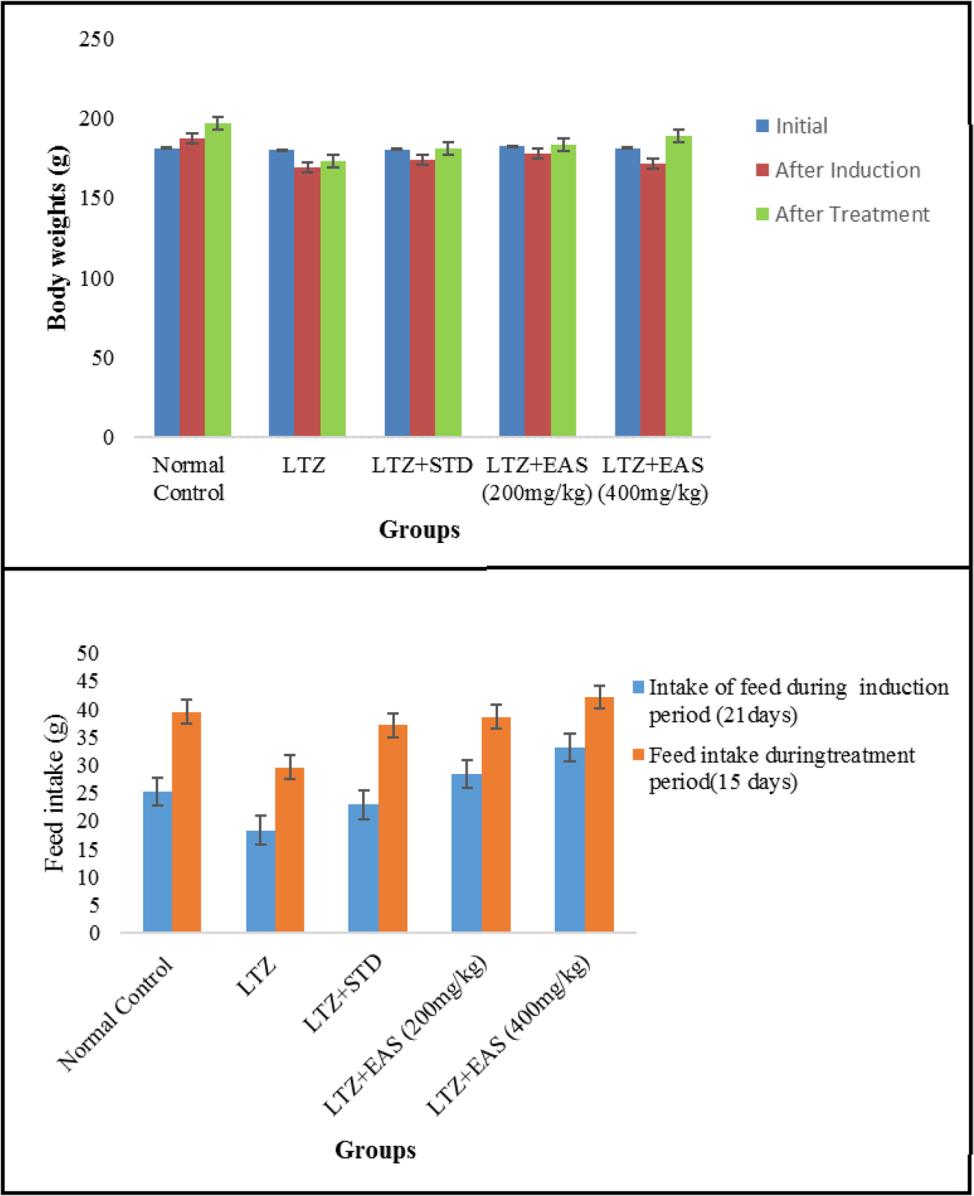
Effect of *Agaricus Subrufescens* on body weights and feed intake in PCOS induced rats

**Fig. 7:**
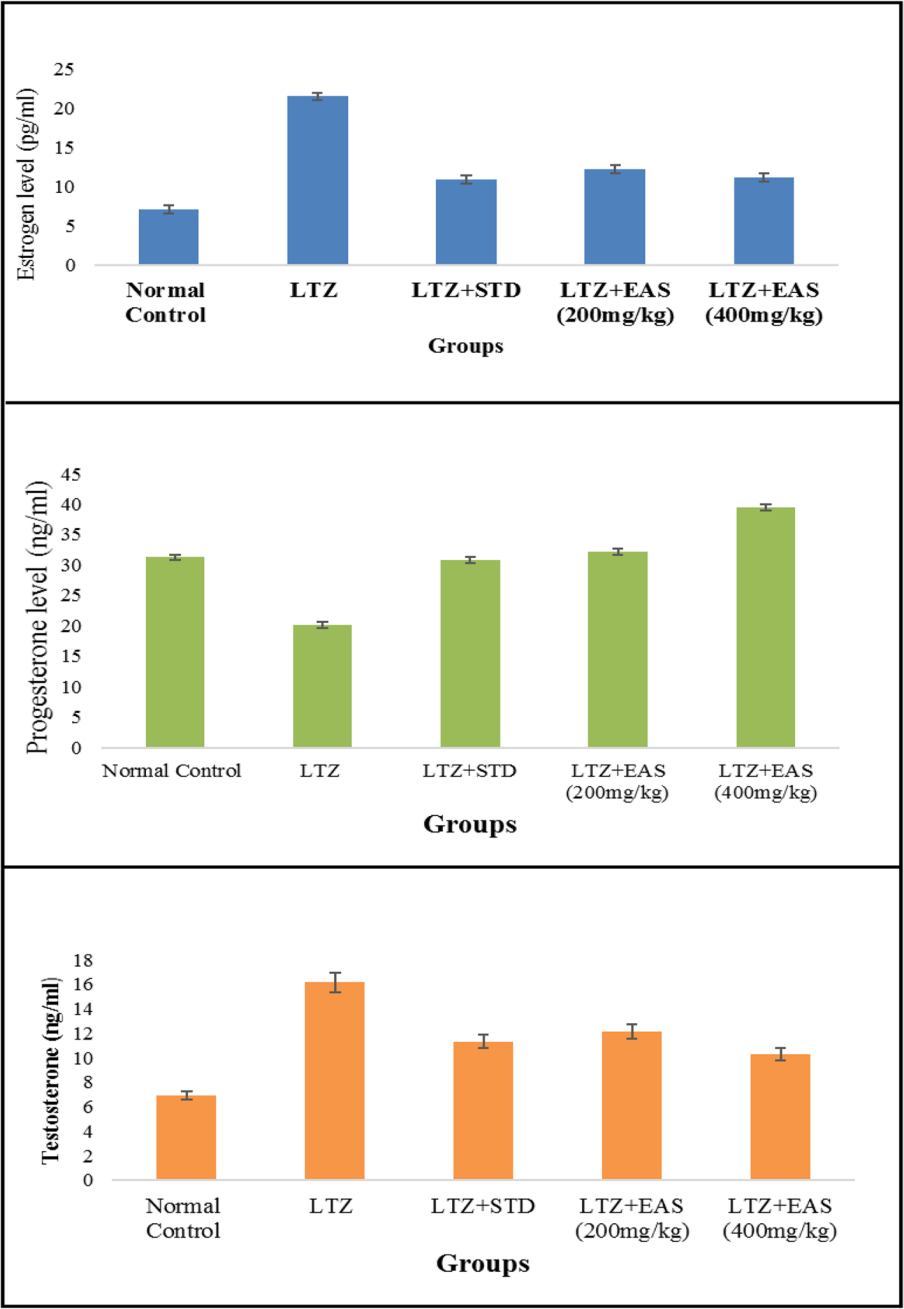
Effect of *Agaricus Subrufescens* on Estrogen, Progesterone and testosterone levels in PCOS induced rat

The oestrogen and testosterone levels of all treatment groups were found to be significantly lower compared to the Letrozole-induced PCOS rats. Anovulation is characterized by the cessation of ovulation due to a decrease in progesterone levels. The control group of individuals with Polycystic Ovary Syndrome (PCOS) exhibited decreased levels of progesterone. The treatment groups exhibited superior performance compared to both the PCOS and normal control groups. The rats with polycystic ovary syndrome (PCOS) that were induced with Letrozole exhibited elevated levels of blood glucose. Letrozole has been shown to augment testosterone production, as well as induce insulin resistance and hyperinsulinemia. The treatment groups exhibited a greater reduction in blood glucose levels compared to the control group induced with Letrozole-induced polycystic ovary syndrome (PCOS), whereas the group undergoing natural recovery did not demonstrate such a reduction. Rats with polycystic ovary syndrome (PCOS) induced by Letrozole exhibited increased levels of total cholesterol and triglycerides. Elevated levels of certain factors have been found to potentially contribute to the development of obesity and cardiovascular disease. *Agaricus subrufescens* exhibited a significant reduction in cholesterol and triglyceride levels across all treatment groups (Groups III, IV, and V) when compared to the control group induced with Letrozole-induced polycystic ovary syndrome (PCOS). Samples were obtained from all animals over a period of 36 consecutive days. Samples were collected daily at 10 am. The findings of this study indicate that the consumption of *Agaricus subrufescens* is associated with a reduction in renal function impairment, as evidenced by a decrease in levels of serum urea and creatinine. The inhibition of hepatic synthesis, promotion of ovarian follicle immaturity, and elevation of androgen secretions in the Letrozole-induced polycystic ovary syndrome (PCOS) rat model result in an increase in both liver and ovary weight. Figures 2, 3, 4 and 5, Tables 2 and 3, Fig. 6, Table 4, Fig. 7, Table 5.

**Table 2:** Effect of various treatments on Body weights in Letrozole induced PCOS rats

**Group**	**Initial**	**After Induction**	**After Treatment**
Normal Control	181.2 ± 1.43	187.1 ± 0.40	196.6 ± 3.38
LTZ	180.2 ± 2.26	169.4 ± 3.20	173.1 ± 3.24
LTZ + STD	180.6 ± 0.378	173.8 ± 2.35	181.3 ± 2.34*
LTZ + EAS (200 mg/kg)	182.6 ± 3.21	178.2 ± 3.25	183.5 ± 4.32**
LTZ + EAS (400 mg/kg)	181.1 ± 3.41	171.5 ± 4.57	189.2 ± 4.21**

The above Values are expressed as Mean ± SEM, *n* = 6.used one way ANOVA to calculate statistical significance of various groups at *P < 0.05,**P < 0.01 by using Dunnette multiple comparison test

**Table 3:** Effect of various treatments on Feed intake in Letrozole induced PCOS rats

**Groups**	**Intake of feed during induction period (21 days)**	**Feed intake during treatment period (15 days)**
Normal Control	25.28 ± 1.45	39.51 ± 1.15
LTZ	18.36 ± 1.12	29.56 ± 1.21
LTZ + STD	22.88 ± 1.13	37.09 ± 1.41
LTZ + EAS (200 mg/kg)	28.32 ± 1.21	38.58 ± 1.33*
LTZ + EAS (400 mg/kg)	33.15 ± 1.12	42.12 ± 1.15*

Values are expressed as Mean ± SEM, *n* = 6.used one way ANOVA to calculate statistical significance of various groups at,* *P* < 0.001 by using Dunnett multiple comparison test

**Table 4:** Effect of various treatments on Hormones in Letrozole induced PCOS rats

**Groups**	**Estrogen (pg/ml)**	**Progesterone (ng/ml)**	**Testosterone (ng/ml)**
Normal Control	7.05 ± 0.25	31.24 ± 1.12	6.91 ± 0.61
LTZ	21.45 ± 0.43	20.1 ± 1.46	16.17 ± 1.02
LTZ + STD	10.92 ± 0.46**	30.80 ± 1.42*	11.32 ± 0.16**
LTZ + EAS (200 mg/kg)	12.19 ± 0.17**	32.1 ± 0.94*	12.12 ± 0.90**
LTZ + EAS (400 mg/kg)	11.15 ± 0.54**	39.52 ± 1.25**	10.26 ± 0.65**

The above Values are expressed as Mean ± SEM, *n* = 6.used one way ANOVA to calculate statistical significance of various groups at,*P < 0.01,**P < 0.001 by using Dunnette multiple comparison test

**Table 5:** Effect of various treatments on Blood Glucose in Letrozole induced PCOS rats

**Groups**	**Initial Blood Glucose**	**Blood Glucose after Induction**	**Blood Glucose after Treatment**
Normal Control	112.5 ± 2.51	120.2 ± 2.574	107.6 ± 3.66
LTZ	113.3 ± 2.12	196.6 ± 7.46	172.3 ± 4.24
LTZ + STD	108.7 ± 3.26	193.2 ± 1.24	138.6 ± 7.51***
LTZ + EAS (200 mg/kg)	108.6 ± 3.24	198.5 ± 2.11	161.5 ± 4.45*
LTZ + EAS (400 mg/kg)	113.6 ± 2.13	188.8 ± 3.62	154.2 ± 2.41**

Values are expressed as Mean ± SEM, *n* = 6.used one way ANOVA to calculate statistical significance of various groups at*P < 0.05,**P < 0.01,***P < 0.001 by using Dunnette multiple comparison test

### Biochemical parameters

**Fig. 8:**
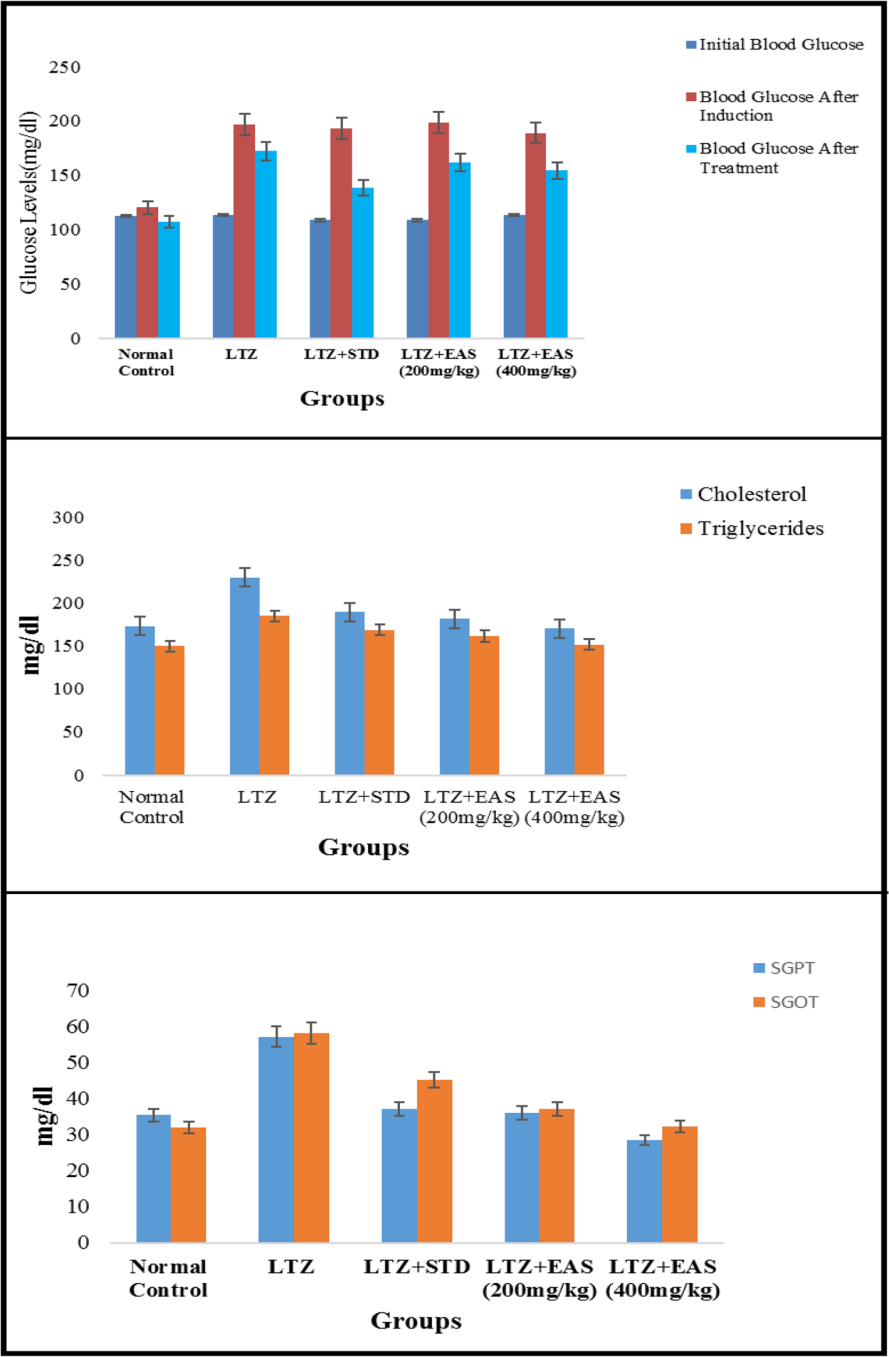
Effect of *Agaricus Subrufescens* on blood glucose level; Cholesterol & Triglycerides; SGPT & SGOT levels in PCOS induced rats

**Fig. 9:**
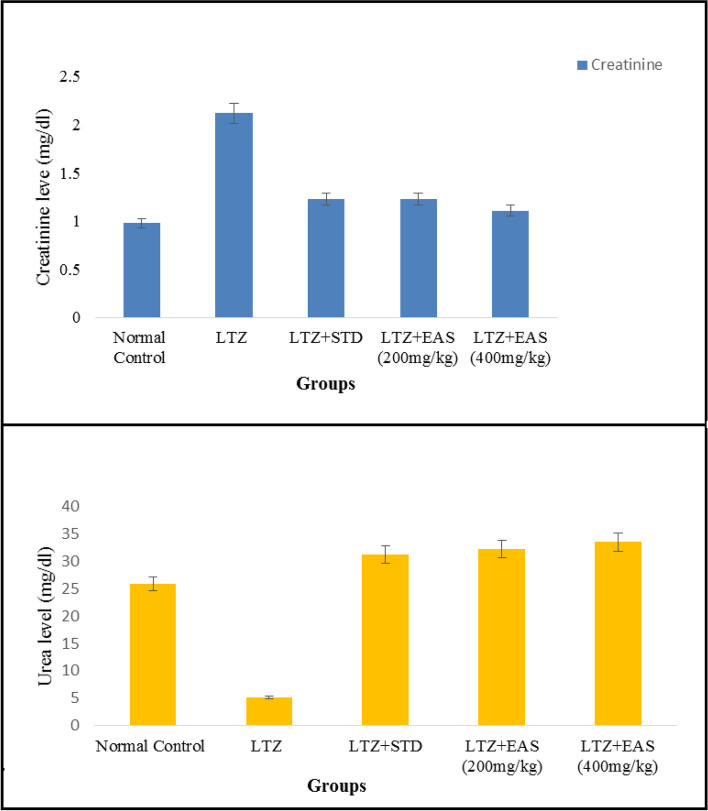
Effect of *Agaricus Subrufescens* on Creatinine and Urea levels in PCOS induced rats

**Fig. 10:**
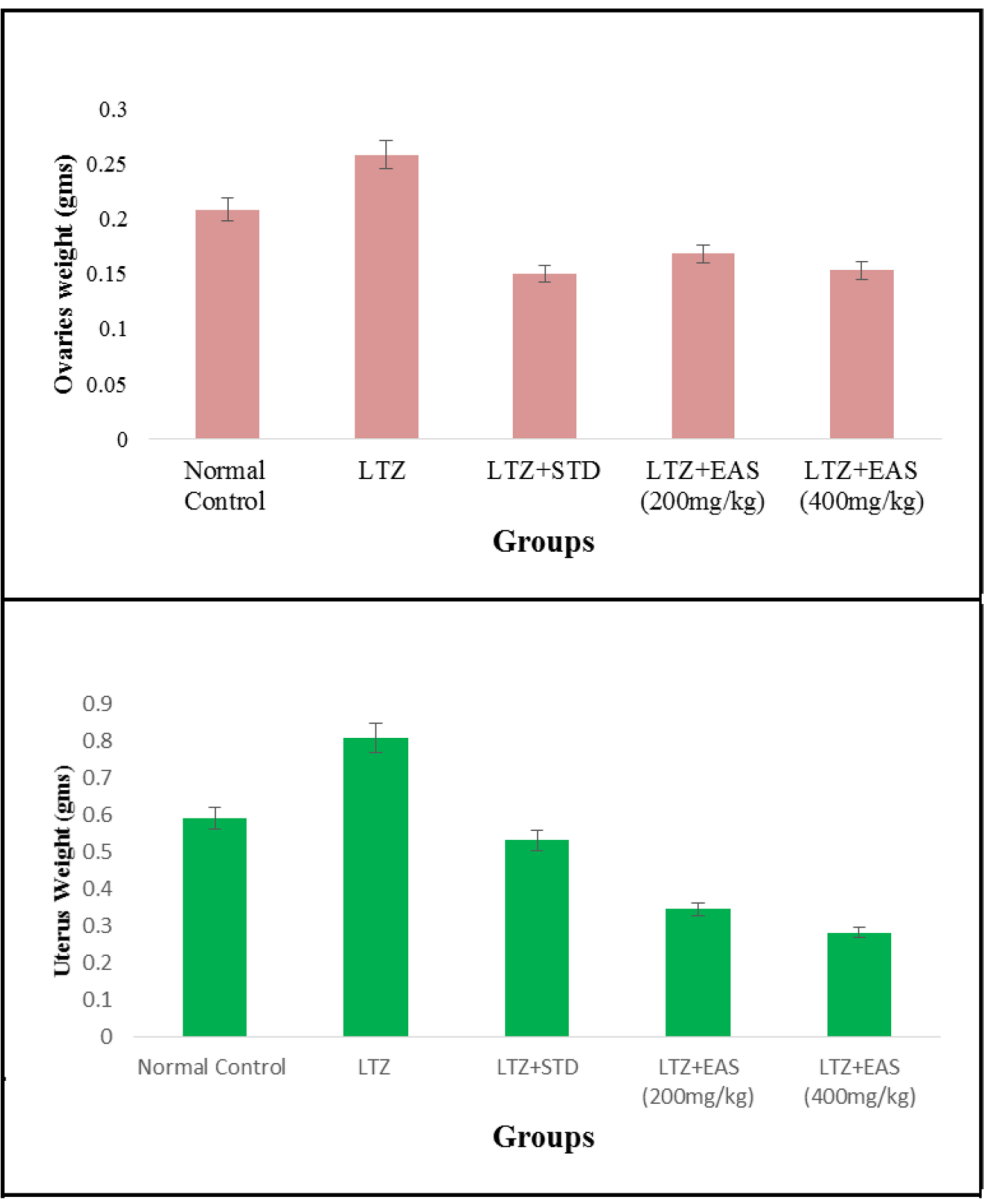
Effect of *Agaricus Subrufescens* on Ovaries and Uterine weights in PCOS induced rats

**Fig. 11:**
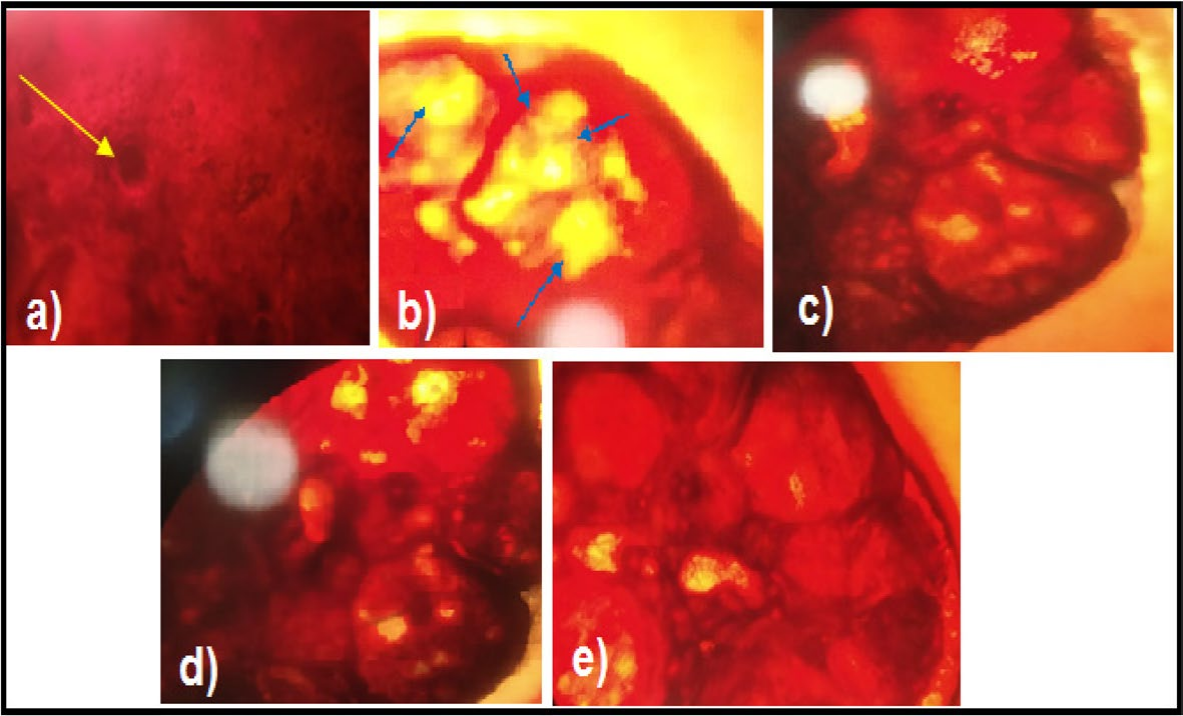
Histopathology observation a) Normal control b) Letrozole induced animals c) Letrozole + clomiphene citrate (standard) d) EAS 200 mg/kg e) EAS 400 mg/kg

Tables 6 and 7, Fig. 8, Table 8, Fig. 9, Table 9, Figs. 10 and 11.

**Table 6:** Effect of various treatments on cholesterol and triglycerides in Letrozole induced PCOS rats

**Groups**	**Cholesterol (mg/dl)**	**Triglycerides (mg/dl)**
Normal Control	172.6 ± 2.27	149.6 ± 2.34
LTZ	229.4 ± 9.52	184.6 ± 4.31
LTZ + STD	189.5 ± 6.51*	168.4 ± 1.32*
LTZ + EAS (200 mg/kg)	181.5 ± 2.48*	161.2 ± 2.12**
LTZ + EAS (400 mg/kg)	170.2 ± 5.91**	151.5 ± 1.09**

Values are expressed as Mean ± SEM,*n* = 6.used one way ANOVA to calculate statistical significance of various groups at*P < 0.05,**P < 0.01,by using Dunnette multiple comparison test

**Table 7:** Effect of various treatments on SGOT and SGPT levels in Letrozole induced PCOS rats

**Groups**	**SGPT (mg/dl)**	**SGOT (mg/dl)**
Normal Control	35.41 ± 1.12	32.10 ± 1.17
LTZ	57.25 ± 4.23	58.24 ± 3.54
LTZ + STD	37.12 ± 2.42*	45.28 ± 2.38*
LTZ + EAS (200 mg/kg)	36.04 ± 1.52***	37.25 ± 1.24*
LTZ + EAS (400 mg/kg)	28.52 ± 1.34 ***	32.24 ± 1.21 *

Values are expressed as Mean ± SEM, *n* = 6 used. one way ANOVA to calculate statistical significance of various groups at*P < 0.05,**P < 0.01,***P < 0.001 by using Dunnette multiple comparison test

**Table 8:** Effect of various treatments on Creatinine and Urea in Letrozole induced PCOS rats

**Groups**	**Creatinine (mg/dl)**	**Urea (mg/dl)**
Normal Control	0.98 ± 0.03	25.83 ± 1.13
LTZ	2.12 ± 0.344	5.17 ± 1.22
LTZ + STD	1.23 ± 0.06*	31.24 ± 0.92**
LTZ + EAS (200 mg/kg)	1.23 ± 0.07*	32.23 ± 1.61**
LTZ + EAS (400 mg/kg)	1.11 ± 0.08**	33.44 ± 0.71*

The above Values are expressed as Mean ± SEM,*n* = 6.used one way ANOVA to calculate statistical significance of various groups at*P < 0.05,**P < 0.01,***P < 0.001 by using Dunnette multiple comparison test

**Table 9:** Effect of various treatments on weights of Ovaries & Uterus in Letrozole induced PCOS rats

**Groups**	**Ovaries**	**Uterus**
Normal Control	0.208 ± 0.001	0.592 ± 0.03
LTZ	0.256 ± 0.02	0.806 ± 0.04
LTZ + STD	0.151 ± 0.01*	0.531 ± 0.03**
LTZ + EAS (200 mg/kg)	0.169 ± 0.01***	0.346 ± 0.02
LTZ + EAS (400 mg/kg)	0.155 ± 0.01*	0.282 ± 0.03***

Values are expressed as Mean ± SEM,*n* = 6.used one way ANOVA to calculate statistical significance of various groups at*P < 0.05,**P < 0.01,***P < 0.001 by using Dunnette multiple comparison test

## Conclusion

This study found that the administration of oral Letrozole was associated with the development of polycystic ovarian disease. The results indicated heightened levels of blood glucose, total cholesterol, and triglycerides, as well as alterations in hormone levels such as increased testosterone and estrogen and decreased progesterone. Additionally, menstrual irregularities were corroborated by examining vaginal smears and observing histopathological changes in the ovaries of the control group with polycystic ovarian disease. The treatment groups of *Agaricus subrufescens* exhibited reductions in blood glucose levels, total cholesterol levels, and testosterone levels. The presence of phytoconstituents such as glycosides, sugars, alkaloids, saponins, terpenoids, proteins, steroids, and phenolic substances may be responsible for this phenomenon. The consumption of *Agaricus subrufescens* was found to result in a reduction in blood glucose levels, testosterone production, as well as a decrease in anovulation and monthly irregularity. In order to enhance the treatment and management of polycystic ovarian disease, it is imperative to undertake active constituent isolation and comprehensive clinical investigations. This study found that the levels of SGOT and SGPT in the control group with polycystic ovary syndrome (PCOS) were significantly elevated compared to the levels observed in the normal control group. The findings indicate the presence of hepatic dysfunction associated with polycystic ovary syndrome (PCOS). A decrease in levels of SGOT, SGPT, and ALP was observed across all treatment modalities. The presence of heightened levels of serum urea and creatinine in Letrozole-induced polycystic ovary syndrome (PCOS) rats can potentially lead to renal impairment. The process of atherogenesis initiates with the dysfunction of endothelial cells. The findings of this study indicate that the consumption of *Agaricus subrufescens* is associated with a reduction in renal function impairment, as evidenced by a decrease in levels of serum urea and creatinine. The inhibition of hepatic synthesis, promotion of ovarian follicle immaturity, and elevation of androgen secretions in the Letrozole-induced polycystic ovary syndrome (PCOS) rat model result in an increase in both liver and ovary weight. The weight of the endocrine organs decreased in all treatment groups. *Agaricus subrufescens* may potentially benefit from this intervention. The histopathological examination of the control group in the study on polycystic ovary syndrome (PCOS) revealed a higher presence of cysts and theca lutein cells compared to the groups receiving therapy. The treatment groups exhibited significantly reduced cyst numbers compared to the PCOS control animals.

## Data Availability

The datasets/information used for this study is available on reasonable request.

## Abbreviations

PCOS: Polycystic Ovarian Syndrome
CMC: Carboxy methylcellulose
LTZ: Letrozole
SGOT: Serum Glutamic Oxaloacetic Transaminase
SGPT: Serum Glutamic Pyruvic Transaminase
*SHBG*: *Sex hormone binding globulin*
LH: Luteinizing Hormone
FSH: Follicle Stimulating Hormone
DHEA: Dehydroepiandrosterone
DHEAS: Dehydroepiandrosterone sulphate
EAS: *Agaricussubrufescens* Extract
GnRH: Gonadotropin releasing hormone
